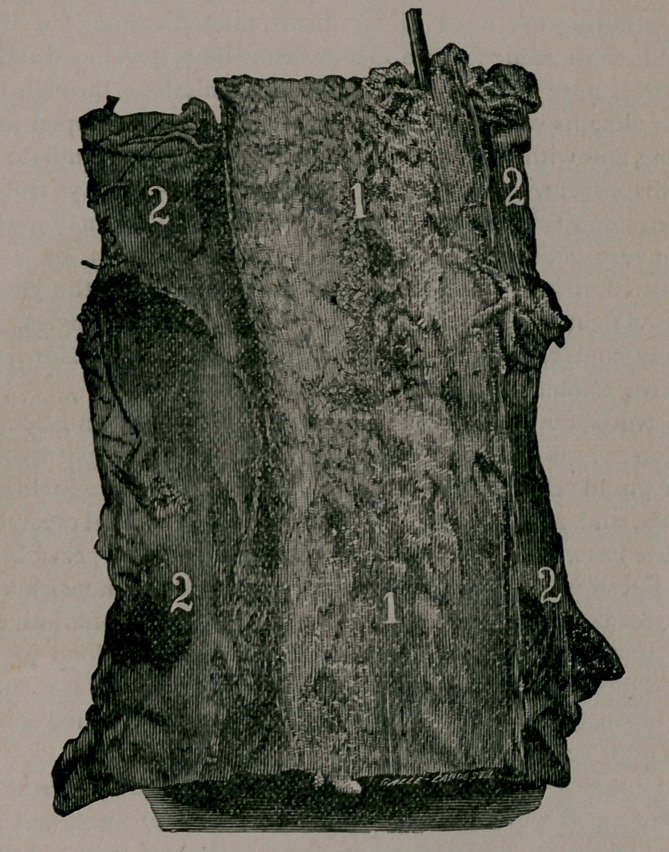# Gunshot Fractures of the Spinal Column, with Report of Three Cases*This article was in competition for the prize offered by the T. S. M. A.

**Published:** 1886-09

**Authors:** W. J. Burt

**Affiliations:** Austin, Texas


DANIEL'S

### Medical ★ Journal,

PUBLISHED MONTHLY AT

AUSTIN, TEXAS.

Vol. 2.] SEPTEMBER, 1886. [No. 3.

"*Scribimus indocti, doctique!*"

### Original Articles.

Contributed Exclusively to this Journal.

*The Articles in this Department are accepted, and published with the understanding that we are not responsible for, nor do we indorse the views and opinions of the writers, by so doing.*

### *GUNSHOT FRACTURES OF THE SPINAL COLUMN, WITH THE REPORT OF THREE CASES.

*By W. J. Burt, M. D., Austin, Texas.*

[For Daniel's Texas Medical Journal.]

GUNSHOT fractures of the vertebræ are usually comminuted and compound injuries. They are frequently, also, complicated with grave lesions of the abdominal and thoracic viscera, or of the great vessels of the neck, and prove immediately fatal before there is opportunity for observation or treatment.

For descriptive purposes these fractures may be divided into two classes, viz: those restricted to the apophyses and those involving the bodies of the vertebræ. In either class the spinal cord may, or

*This article was in competition for the prize offered by the T. S. M. A.

may not be injured. Injuries to the cord, from either the missile or fragments of bone, are essentially contused and lacerated in their nature. Fractures of the vertebræ—body or apophyses—are always of a serious nature, and prove fatal in a large per cent of cases, either from the primary injuries, or from secondary lesions.

While it is true, in at least fifty per cent of the cases, that the damage to the vertebræ and cord is beyond the help of the surgeon, yet there are cases of fractures of the processes or laminæ, or even the pedicles, with or without compression of the cord, that may be relieved by prompt surgical attention.

The desire of the writer is to collate some facts and statistics in the history of this class of fractures, and to suggest a treatment for some of them, that will, it is believed, encourage surgeons to treat more actively and promptly, according to clear and accepted surgical principles, what some authors mention, *mildly*, as a possible treatment.

HISTORY.

In the history of the British army in the Crimea, thirty-three cases of gun shot fracture of the vertebræ are reported among the British soldiers. Twenty-nine cases, or ninety per cent, proved fatal. The four cases of recovery—two officers and two men—were "mere fractures of the processes of the cervical vertebræ."

In the French army in the Crimea, there were reported one hundred and ninety-four cases of these fractures, with 181, or 93 per cent of deaths.

In the war between Prussia and Hanover, in 1866, the mortality, in these injuries, was 75 per cent.

But the most reliable and interesting statistical history of these fractures is furnished by Drs. Barnes & Otis, in *The Medical and Surgical History of the War of the Rebellion.*

In Circular No. 6, S. G. O., 1865, it is reported that of 187 recorded cases, 180, or 95 per cent, proved fatal. Of the 7 cases which did not die from the injuries, according to the circular, "6 were fractures of the apophyses only." One was a fracture of the body of the third cervical vertebra.

Drs. Barnes & Otis have tabulated several hundred cases, which are here presented :

TABLE NO. 1.

*Report of 642 Cases of Gun Shot Fracture of the Vertebræ—Military Surgery.*

| Region. | No. of Cases. | No. Died. | No. Discharged. | No. R'td to Duty. | Unkno'n. | Per Cent Mortality. |
|---|---|---|---|---|---|---|
| Cervical | 91 | 63 | 19 | 8 | 1 | 70 |
| Dorsal | 131 | 87 | 32 | 18 | .. | 63.5 |
| Lumbar | 149 | 66 | 51 | 28 | 4 | 45.5 |
| Cervico-Dorsal | 2 | 1 | 1 | .. | .. | 50 |
| Dorso-Lumbar | 3 | 3 | .. | .. | .. | 100 |
| Not stated | 260 | 129 | 72 | 50 | 9 | 51.4 |
| Total | 642 | 349 | 175 | 104 | 14 | 55.5 |

Prof. John A. Liddell says, "doubtless these 279 cases, marked 'discharged and returned to duty,' were mere fractures of the transverse or spinous processes."

For the purpose of comparison, the following table, as compiled by Dr. Otis in 1867, is presented. These cases, or nearly all of them, are from the records of civil surgery:

TABLE NO. 2.

*Report of 394 Cases of Gun Shot Fractures of the Vertebræ—Civil Surgery.*

| Region. | Cases. | Deaths. | Not Improved. | Recov'd. | Per Cent Mortal'ty |
|---|---|---|---|---|---|
| Cervical | 212 | 164 | 10 | 38 | 77.3 |
| Dorsal | 130 | 82 | 20 | 28 | 63.1 |
| Lumbar | 57 | 34 | 8 | 15 | 59.7 |
| Not stated | 19 | 5 | 6 | 8 | 26.3 |
| Total | 418 | 285 | 44 | 89 | 68.2 |

In 24 of the above cases the cervico-dorsal, or dorso-lumbar vertebræ, were fractured, and are accredited, in the table, to both regions; hence the cases aggregate 418 instead of 394 cases.

Table No. 1, of 642 cases, in military surgery, gives an average mortality, where the region was noted, of 60 per cent; Table No. 2, in civil surgery, of 393 cases, where the region was noted, a mortality of 70 per cent. According to the first table, we find that the lumbar vertebræ are more frequently fractured, next the dorsal, and last in point of frequency the cervical. While according to the second table—civil cases—the frequency of the injuries to the different regions is reversed. The cervical first, the dorsal next, and lumbar last. Or, to state it differently—in military surgery 25 per cent of fractures were cervical, 35 per cent dorsal, and 40 per cent lumbar; while in civil surgery 53 per cent were cervical, 32 per cent dorsal, and 15 per cent lumbar.

Drs. Barnes & Otis sum up their observations of reported cases as follows, viz:

" Cases of gun shot injuries to the vertebræ were commonly fatal; a few cases, where the apophyses only were injured, and fewer still where the bodies were fractured, made partial recoveries."

Of the 5 cases of partial recoveries reported by them, of fractures of the cervical vertebræ, in three of them it was definitely known that the spinous processes, only, were injured; in one case, a transverse process; and in one very remarkable case, the body of the third cervical was fractured. I give a brief report of the case as it is presented, officially, in a recent work on surgery.

July 2, at the battle of Gettysburg, a soldier was wounded by a conical ball, which passed through the upper lip, below the soft palate, and penetrating the posterior pharynx, lodged in the body of the third cervical vertebra, resulting in paralysis of the upper and lower extremities. Thirty-six days afterwards the ball was removed and the paralysis gradually disappeared. Eight months from the date of the injury, Dr. W. W. Keen, jr., at Turner's Lane hospital, Md., uses the following language about him : "Nearly the entire body of the third cervical vertebra has come away, including the anterior half of the transverse process, and the vertebral foramen."

Now what the doctor means by the "anterior half of the transverse process coming away" I cannot comprehend ; and when he states that the "vertebral foramen" came away, my credulity is overtaxed. If, for instance, the doctor had reference to the "foramen" for the vertebral artery "coming away," he should have informed us of the behavior of the artery. If, however, he had reference to the "foramen" for the spinal cord "coming away," his indifference to the cord, in its new surroundings, will always bring upon him the criticism of every true investigator in this department of surgery.

As curiosities in surgical literature and to, show the tenacity of life when the spinal cord and colum are greatly mutilated, I here present, in synopsis, a few cases that occurred and were officially reported during the recent war in the United States :

Case 1—Chas. S., 87th Pennsylvania, was wounded July 9, 1864, and died October 13, 1864. At the autopsy the ball was found lodged in the spinal canal of the 10th dorsal vertebra, and yet the man lived 96 days.

Case 2—T. K., 6th United States cavalry, was wounded by a 36-calibre pistol ball March 26, 1866, and died 63 days afterwards. The ball had penetrated the 3rd lumbar vertebra, entered the canal and lodged in the canal of the 4th lumbar.

Case 3—Michael X., 13th New York, was wounded June 27, 1862, and died December 27, 1862. He lived 7 months with the ball lodged in the spinal canal of the 5th lumbar vertebra. There was no paralysis and but little inconvenience from the ball for 3 months and 20 days.

Case 4—C. S., wounded July 9, 1864, by a musket ball, which was found to have entered the spinal canal of the 8th dorsal vertebra and passed up through 7th, 6th and into the 5th dorsal, destroying the cord and meninges, and the man lived 3 months and 4 days.

Case 5—A. McLain, shot by a canoidal pistol ball May 11, 1863. The ball entered the spinal canal cf the 8th dorsal and passed up the canal and lodged in the canal of the 1st cervical. According to this history the ball traversed the spinal canal of 16 vertebræ and destroyed, or seriously damaged the cord and meninges, and yet the man lived 8 days.

I now call attention to three cases of G. S. fracture of the spinal column, which have recently been observed in this city by the writer.

Alice Davis, colored, aged 26, was wounded by a 45-calibre pistol ball October 28, 1885. The ball entered her body, while standing, three inches to the right of the spinal column, and opposite the 8th dorsal vertebra ; it passed toward the 8th dorsal and through the laminæ and base of the spinous process of that bone, and entering the abdominal cavity, lodged between the 8th and 9th ribs of the left side, from which point it was removed. The women fell to the floor, completely paralyzed in her lower extremities, having neither sensation nor motion in either leg, the paralysis showing that the functions of the white substance of the posterior columns and the gray central matter of the cord were arrested or destroyed. Complaint was made of sharp pains in the track of the ball and a tingling sensation in the upper extremities. Retention of urine and fœces for six days. After which the discharges were involuntary. On November 2, five days after the injury, a large gangrenous slough appeared over the sacro-lumbar region, about 6x5 inches in extent. Several smaller ones showed themselves about the same time over the heels, instep and gastrocnemii. There were neither pain nor discomfort in these sloughing surfaces. They could not have resulted from pressure alone. Some other element as a potent factor must have existed. A reasonable explanation is that from some inhibitory influence, there was persistent contraction of the blood vessels supplying the parts, and by these means, cutting off the nutrition, which, necessarily, resulted in sphacelus. Another point, confirmatory of the position, lies in the fact of the low vitality of the ulcerated or raw surfaces ; for there was neither pains, sensation, nor any disposition to granulation in these surfaces and almost no blood flowed from handling them. A high septicæmic fever of 104 to 105 °, with delirium, and great prostration developed on the 7th day of the injury and continued, with occasional remissions, during life. On the 11th of November paralysis of the upper extremities, and a comatose condition, were observed, and they continued until death on the 16th of November, being 19 days from the date of injury.

Autopsy.—The bullet had passed through the laminæ and base of the spinous process of the 8th dorsal, forcing spiculæ of bone against the meninges and cord, lacerating the former, and contracting the canal to about half its normal size.

Several clots of blood were within the canal, external to the theca. Osteo-myelitis of the 7th and 8th Dorsal, with partial destructive degeneration of the intervertebral substance was noted. The dura mater was highly inflamed, and adhered in several places to the cord and walls of the canal. On making a vertical section of the cord in these bones, the gray matter was found softened, and the incised surfaces of the cord had a worm-eaten appearance for about 3 inches in length. The nerves originating in this part of the cord, on being opened longitudinally, showed evidences of inflammation, and in their center, in a small degree, there was this characteristic softened and worm-eaten appearance, and in places, a slight tinge of red was observed.

CASE NO. II.

Nathan Elgin, colored, aged 28, wounded by a 45 calibre pistol ball February 8th, 1886, at 10 P. M. The ball entered the left side or back about 3½ inches from the spinal column, and 4 inches above the crest of the left Ilium, and passed towards the first lumbar vertebra. Paralysis of the lower limbs came on at once, with retention of urine and fœces. He complained of much pain in the lumbar region, and was entirely rational. Drs. Graves and Cummings slightly enlarged the orifice of entrance and ascertained the ball had entered the spinal column, and could not be found. They gave an unfavorable diagnosis, which was not satisfactory to the friends of the wounded man, and two homœopathic physicians were called to take charge of the case.

At 9 A. M. February 10th, 35 hours after the injury was received Elgin's condition was unchanged. He was quiet, rational, with no fever, and but little suffering.

The writer was informed that the homœopathic attendants cut down upon the rib at the orifice of entrance and removed a piece of bone. The patient never rallied and only spoke one time after this operation, and died at 11 P. M. There is no doubt but his death was hastened by this surgical interference. The depressing effects of he anæsthetic, and loss of blood, and the shock of the operation were potent factors in terminating life so quickly. The heart's action was not disturbed until the heroic effort to remove a few inches of costal bone had been planned and executed.

Autopsy, 12 hours after death. Parts of the small bowels, and the descending colon were congested and inflamed. The left renal vein and the ascending vena cava were filled with clotted blood. The left psoas muscles were much swollen and congested, as was the peritoneum in the vicinity. The left kidney was swollen, engorged and softened, as was also the right one, but less so. The ball had passed in the rear of the psoas muscles, left kidney and peritoneum, and declining slightly downwards, had entered the inter-vertebral foramen, between the last dorsal and first lumbar vertebræ, and passed into the canal, and lodged in it, partly in the first, and partly in the second lumbar, fracturing the pedicles, and articulating surfaces of both bones. The cord and meninges in the track of the ball were almost totally destroyed. The bullet, in its passage reversed ends, and was found with the conical end pointing towards the channel of entrance.

CASE NO. III.

Sam. Pearson, colored, aged 27, was wounded November 25, 1885, by a 38 calibre pistol ball. The ball entered about 4 inches to the right of the seventh dorsal vertebra and passed directly towards the spinal column, producing paralysis of the lower limbs, abdominal muscles and pelvic viscera. There was retention of urine and fœces for about 2 weeks, and after that time, the discharges were involuntary, the urine continuously dribbling away. On November 28, a second examination was made, under the influence of chloroform and a free incision about 3 inches in length was made by Dr. Swearingen along the right side of the spinous processes of the 7th, 8th and 9th dorsal vertebræ, and the incision connected with the track of the ball. The location of the ball was not ascertained. A perforated rubber tube was carried along the track and incision, and retained for drainage and for mercuric bichloride washes. The wound healed nicely and rapidly. On the 8th of December a large slough occurred over the sacrum, the left trochanter major and head of the left femur, involving the capsule, allowing the head to be disarticulated when the limb was moved. Septicæmic fever, about the same time, developed and continued, with more or less intermission and remissions, through the case. Other sloughs of various sizes, on other parts, developed from time to time, and with no kind of treatment would they granulate or show any disposition to resolution. January 12th,—48 days from injury, jerkings and twitchings of lower limbs and abdominal muscles became very annoying. They came in paroxysms—3 or 4 times a day—with distressing nausea. As time progressed, the jerkings became violent and would flex the limbs at right angles, but were usually controlled with opiates. There has been a slow, but progressive emaciation throughout the case.

Notwithstanding complete paralysis of motion and sensation in the lower limbs, occasional paroxysms of muscular spasms occurred, that would completely flex the lower limbs, producing intense nausea, and great prostration. The intellect remained clear, throughout the case, except a few times while a high fever prevailed. Gastric digestion was good, and a genuine relish for nourishment, and enjoyment of food were very noted. But the assimilative organs were not competent to appropriate this digested food into blood, hence the progressive emaciation. He was really "skin and bones and ligaments" when death closed the scene. He died quietly 154 days after receiving the wound.

An autopsy was held. The pistol ball entered the ninth dorsal vertebra at the base of the right transverse process, and passed a little downward, and lodged in the spinal canal in that bone, completely imbedding itself in the meninges and spinal cord. There were also small spiculæ of bone penetrating into the cord around the ball. The meninges were thickened, and bore evidences of inflammatory deposits.

The cord itself was softened, but partly intact in this locality. With this amount of damage to the cord and meninges, and the ball and spiculæ of bone producing constant pressure and irritation, resulting in softening of the cord, and producing in it inability to transmit sensory cr motor impressions, it is very remarkable that such a case should live 154 days.

In these three cases reported, the damage to the spinal column, cord and meninges, was so great, that no treatment or operation could have relieved them.

### DIAGNOSTIC SYMPTOMS.

These fractures being compound in their nature, can usually be diagnosed by exploring the wound with the finger, or a suitable probe. Simple fractures of the spinous processes or laminæ may generally be recognised by pain, and tenderness under pressure, and by increased mobility and crepitation when the processes are moved. Fractures of the pedicles and bodies of the vertebræ are more obscure and almost always involve the cord, when paralysis of the limbs and sphincters follows; also priapism, and afterwards neuropathic sloughs. These symptoms may arise from concussion as well as compression. Whatever arrests the transmission of sensory impressions through the gray matter of the cord, or of motor impressions, through the posterior white columns, may produce paralysis. If the paralysis occurs immediately after the injury, the cause will generally be concussion, compression, or partial, or entire destruction of the continuity of the cord.

If the paralysis comes on some hours afterwards, it is generally due to extravasated blood within the "theca-vertebralis;" when paralysis comes on at a later period, it is often caused by meningitis or myelitis. By these symptoms a diagnosis can generally be made in reference to a fracture, and to the extent of the damage.

But I call attention to another, and so far as I know, a new means or method, that can be practiced, to clear up some obscure cases of injury to the bodies of the vertebræ. I mean an examination after "Simon's method" of rectal exploration, that I now call the "intra-abdominal explorations per rectum," which, as is readily seen, is an extending of the "Simon method" to an exploration of the abdomen, and a digital examination of the anterior portion of the bodies of the lower vertebræ. This method of examination is to be accomplished as follows:

Anæsthetize the patient, paralyse the sphincters, and gently introduce the fingers and hand into the rectum and push its folds, and those of the lower portion of the colon upward, as you would push the finger of a glove into the body of the glove by pressure upon its tip. The fingers are then sufficiently free to examine the bodies of the lower vertebræ, and if they are fractured, the injury may be discovered. This method of examining the bodies of the lower portion of the spinal column I claim as a new departure, or rather a suggestion, that so far as I know, has never been made. Dr. B. E. Hadra, of Austin, informs me that the idea of exploring the abdominal cavity in this way for the purpose of examining the organs of the abdomen, has been in his mind for some time; and he further states, that he has made the examination in the "living subject." The exploration is not as difficult as would at first thought appear, and can be made with no reasonable expectation of serious results following it. I present this as a new method of diagnosis in fractures of the bodies of the lower portion of the spinal column, and take the position that if the method is put to its full practical and clinical test, a differential diagnosis of injuries and disease of the vertebral organs of the abdomen may be made with as much ease and certainty as we now explore and examine the organs of the pelvis. By way of parenthesis, I will suggest, that by this method of exploration a large field is opened for investigation. This method will give us an almost certain diagnosis in supposed extra-uterine pregnancy. Also in tumors of the abdominal cavity, involving the kidneys, liver, spleen and other organs. And just what possibilities there are in store, from a diagnostic standpoint, by this method of recto-abdominal exploration, the future alone can solve.

TREATMENT.

The two pressing indications to meet in gun shot fractures of the vertebræ are, to extract any foreign bodies, and remove any spiculæ of bone that can be found, and place the body as nearly at rest as can be done, observing cleanliness and antiseptic precautions. The use of opiates, if there is much suffering, and cool compresses, wet with an antiseptic fluid, or, if preferred, dry antiseptic treatment with iodoform, may be required, with attention to the bladder and bowels. The two points, however, to which I desire to call attention in treatment, are the following, viz: Incision, in certain cases, near to the spinous processes, and in certain other cases, to trephining, and removing the cause of compression. In reference to the first, I will say, that if the wound of entrance is three or more inches from the spinal column, and an examination discovers a fractured vertebra, the proper surgical treatment is to make a free incision on one, or even both, sides of the spinous process supposed to be damaged, and connect it with the track of the ball. The reasons for this incision are very apparent to any thoughtful surgeon.

1. That a more careful examination may be made of the injuries received.

2. That foreign bodies, and fragments of bone can be more easily found, and readily removed.

3. That more complete drainage of the parts injured can be accomplished, antiseptic washes better, and more easily, used, collections of pus avoided, and osteo-myelitis prevented.

With all these advantages, resulting, more or less, from the incision mentioned, a neglect to perform it lays the surgeon subject to criticism. The history of many cases reported, in both civil and military surgery, where inflammatory deposits, accumulations of pus, and necrosed bones, have been secondary causes of death, are potent reasons for free openings and thorough drainage. Take an instance where a ball enters six inches from the spinal column, and passes into, or through, some part of it, leaving a track of six inches, through tough fascia, and muscles of the back. Any advice to wash this tortuous track antiseptically, would be a play upon the credulity of a mere tyro in surgery.

The importance of these incisions I desire to impress with all the force and persuasion language can convey. For, if the surgeon fails to perform this primary operation, and allows the secondary lesions to arise, mentioned above, in his patient, and become factors in producing his death, he will not, and cannot, stand blameless before just criticism.

### TREPHINING THE SPINAL COLUMN.

The operation of excising and removing parts of a vertebra by trephine, saw, or chisel and mallet, has been mentioned since the days of Paulus Ægineta, and advocated since the time of Pare as a theoretical possibility.

Occasionally, through the generations, a vigorous flash of originality lights up the annals of surgery, and projects its rays far in advance of what is denominated conservatism; and laparotomy, oophorectomy, enterorrhaphy and such like measures, become fixed operations in surgery. It was the intensity of thought, the burning thirst for excellence, and the combination of peculiar attributes, that made such men as Watt, and Davy and Faraday potential factors in science; and their discoveries brought wealth and knowledge to the nations. It was when surgeons and the whole surgical literature, condemned excision of the knee point, that Henry Park, of Edinburgh, rose majestically above theory, and resected the knee joint of a man, and saved his leg and foot; which operation is to-day, a brilliant memorial of that surgeon.

In 1814, with all precedents against the operation, the celebrated Henry Cline removed by saw, trephine and chisel the arches of the 11th dorsal vertebra which were pressing upon the cord and producing paralysis. The man lived 17 days. The necropsy showed fracture of the 12th dorsal, dislocation forward of the 11th dorsal, and laceration of the cord and membranes. If the cord had not been so seriously damaged, the operation, removing the compression, might have saved the life of the patient.

John Rhea Barton was probably the first American surgeon to attempt the operation. Prof. Liddell says he "has failed to find one completely successful case on record." Malgaigne calls the operation a "desperate and blind one." Prof. Miller, of Edinburgh, says, "The trephine is not to be thought of." Prof. Erichsen, and many other writers, either condemn or remain neutral in reference to trephining. This of course has reference to trephining the vertebræ, when there is bony pressure upon the cord, and has no reference, whatever, to removing loose or detached pieces of bone.

M. Louis in 1762 was reported to have resected a vertebra, but a careful examination of the report of the case shows it was simply removing loose fragments of bone five days after the injury. Dr. Stephen Smith, at Bellevue hospital, New York, resected the laminæ of the 10th dorsal vertebra, which was pressing upon the cord. He removed that part of the bone and relieved the compression, also about four ounces of dark colored blood escaped from the spinal canal. He found the meninges and cord badly injured. and the results bad, for the paralysis steadily crept upward, and finally caused death by asphyxia. (*New York Journal of Medicine*, 1859, pages 87 to 88.)

Dr. Ashhurst has tabulated 43 cases, where the vertebræ have been trephined, or resected with the following results :

31 cases, or 72 per cent., died. 4 cases not benefitted. 4 cases were relieved. 4 cases results not known.

Of the 31 cases marked "died," it is no where asserted in the reports, that any of them would have lived if the operation had not been performed. It is stated, 4 cases, or 9 per cent, were relieved. Then we argue that the operations were justifiable. No one was killed or seriously damaged by the trephining, and 9 per cent. of all cases were relieved. Let us compare results of resections and excisions in other major operations, and note the per cent. of recoveries. Take G. S. wound of the hip joint. Drs. Otis and Huntington report 43 cases of primary, and 60 cases of intermediate excision, with 93 cases, or 95 per cent. of deaths; recoveries 5 cases or 5 per ct. Or to take the total number of primary, intermediate and secondary excisions; we have 171 cases, with 145, or 86.5 per cent. of deaths, and 13.5 per cent. of recoveries, or did not die. It must be noted that these recoveries stand over against the deaths and do not mean that they recovered in the sense of having useful limbs. In G. S. injury of the knee joint Drs. Gurlt and Culberson have collected 144 cases of resection. Results, 111 cases, or 77 per cent. of deaths ; 33 cases, or 23 per cent. of recoveries. It will thus be seen that, according to statistics, the mortality in excisions of the knee and hip joints from G. S. injuries, is about as great as in excisions, or trephining the vertebræ. No surgeon would think of trephining every case to which he is called. But the argument is, that in some cases, not all of them, the operation is not only a justifiable one, but is demanded from sound surgical principles and experience. Every case must rest upon its merits, as weighed by the judgment of the surgeons. Take a fractured vertebra, with parts of the processes, laminæ or pedicles, pressing against the cord, causing paralysis, and finally, degenerative destruction of the cord, and death of the patient. Could any surgeon hesitate to attempt the removal of the compressing cause, any more than if it was the brain being compressed? In examining the records of trephining the vertebræ, I have been astonished to notice that illustrious surgeons have so often waited until inflammatory deposits and softening, had occurred, before they would attempt the operation. If the trephining is to benefit a patient, suffering from compression of the cord by blood, or missile, or bone, it must be done before the cord has lost its integrity. It is a difficult operation, but the bottom fact, the elementary principle, and clinical experience call just as loud and long for relief, as if the operation was a trivial one.

The actual nature of the injury is not always easily told, and there may, or may not be, compression, and no one can say in advance that such an operation will give relief. But the fact is before us ; there is a G. S. injury directed toward the spinal column, a probe, or the finger detects a fracture; there is paralysis below the injured bone, with retention and all the general and special manifestations of damage to the cord by compression or otherwise. The experience of civil and military surgery, all over the world, is, that such a patient will die from the primary injury, or secondary lesions. What will we do ? If he will certainly die without surgical relief can be given, the effort to give that relief by incision and trephining will not kill him. Should we refuse to give him the only chance of saving his life? It is a mortal peril, and demands an extreme remedy. "*Aut Caesar, aut nullus.*"

## Figures and Tables

**Figure f1:**
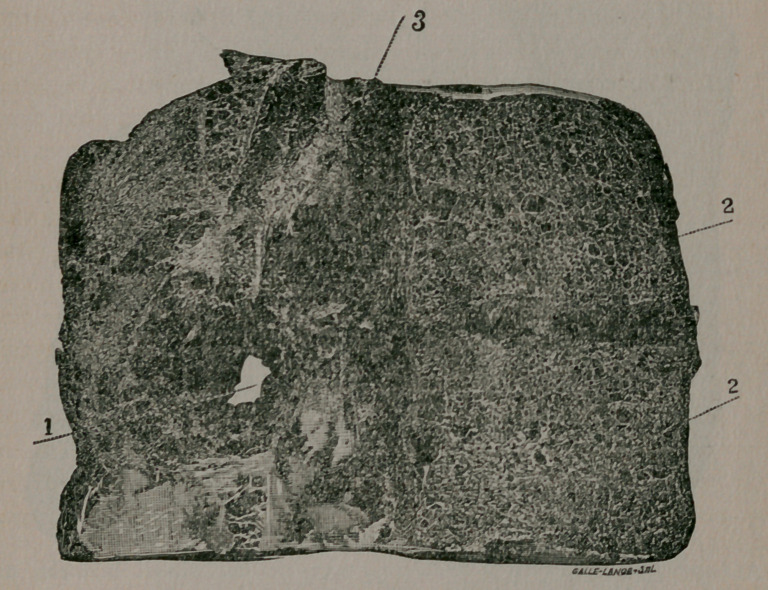


**Figure f2:**
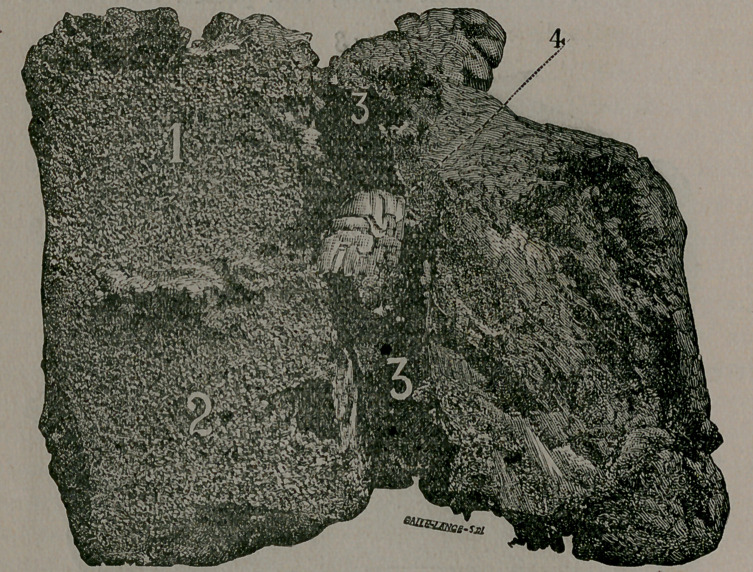


**Figure f3:**