# A Correction

**Published:** 1886-10

**Authors:** B. E. Hadra


### A CORRECTION.

Editor Daniel's, Texas Medical Journal:

In THE article "Gunshot Fractures of the Spinal Column," in the last number of your journal, my name is quoted in conjunction with Simon's method of rectal explorations by the whole hand. It reads there this way, which must necessarily set me in a very queer light, because I neither claim to be a rediscoverer of Simon's method, nor do I consider it to be a procedure unknown to the profession. Though I hate to remonstrate, because of the decease of the writer, I deem it, nevertheless, my duty to defend myself, and to claim for myself what is mine.

The idea of using Simon's method in vertebral injuries struck me first in Garfield's case; later on, about two or three years ago, when a case of that kind was reported and discussed before the Texas Medical Association, I made such a suggestion, which fact can be corroborated by the members then present. A few weeks before the last meeting of the Texas State Medical Association, about February, 1886, Dr. Burt, in accidental conversation, told me about his paper, and I then spoke of my idea. He wishing to use it, I consented to it, provided he would state my authorship, as I intended, and still intend, to put the method into practice, when a suitable case should offer itself. (I would not wonder if it was practiced already by somebody else.) I hope nobody will charge me with vain pugnacity for these lines. Dr. Burt surely would have made such amends himself, if called on. Whether the point in question is of value or not, is of minor importance to me. In writing this I am actuated by a desire to be extricated from what I feel to be a false position before the profession.

B. E. HADRA, M. D.

